# Andes Hantavirus Outbreak on a Cruise Ship, 2026

**DOI:** 10.1056/NEJMc2606496

**Published:** 2026-05-20

**Authors:** Andes Virus Outbreak Working Group

To the Editor:

On the 27th of April 2026, an adult male (case 3) was initially medically evacuated from the Dutch-flagged expedition cruise ship MV *Hondius* to Ascension Island with a severe acute respiratory infection (SARI), reporting shortness of breath and fever that began on the 21st. He had signs of pneumonia, although the chest x-ray (CXR) was unremarkable. His condition worsened and transferred for ventilator support and intensive care in Johannesburg, South Africa (SA)^1^. He was in shock with acute respiratory distress syndrome and an atypical pneumonia picture on CXR. The differential diagnosis in this situation is very broad and includes atypical pneumonias, bacterial or fungal sepsis, and vector-borne diseases such as Malaria or Dengue. A diagnostic evaluation was unrevealing including respiratory pathogen panels, malaria smear and antigen, fungal biomarkers, blood cultures and legionella urinary antigen. Further details are provided in the Supplementary Material.

A virtual consultation was held on the 2nd of May with SA, the United Kingdom (UK) and Dutch counterparts to discuss the potential link to two recent fatalities associated with the same ship. On 2 May, the UK officially reported a cluster of severe acute respiratory illness, including two deaths and one critically ill passenger aboard the cruise ship, of unknown etiology to the World Health Organization through the International Health Regulations (IHR, 2005). The Netherlands did the same through the restricted Early Warning and Response System (EWRS).

The cruise ship departed Ushuaia, Argentina, on 1 April 2026 and followed an itinerary across the South Atlantic. The first fatality (case 1) developed respiratory symptoms on 6 April and died on 11 April on board the ship with SARI and respiratory failure. No microbiological tests were performed. He had a 3-month travel history to Argentina, Chile and Uruguay prior to his departure on the cruise^2^. His body was disembarked at the scheduled stop at Saint Helena on 24 April. His partner (case 2), who also went ashore that day, developed similar symptoms and started a return journey to the Netherlands via Johannesburg on 25 April. She was too ill to complete her connecting flight and died at the emergency department at a Johannesburg hospital. Considering the travel history, negative respiratory results and the rapid progression of cases 2 and 3, the expanded differential diagnosis included Hantavirus Cardiopulmonary Syndrome (HCPS) and samples were sent to the National Institute for Communicable Diseases (NICD) in SA, where the diagnosis was confirmed on 2 May using a pan-hantavirus RT-PCR (assay details in supplement). The antemortem sample from case 2 was traced, and sequencing of the L segment of both cases 2 and 3 at NICD confirmed an ANDV on 6 May (Supplementary Material)^3^.

On 2 May, the cruise ship had a total of 147 individuals on board, including 88 passengers and 59 crew members, from 23 countries. As of 13 May 2026^4^ there are 10 cases (11th subsequently removed), including 3 deaths and a case fatality rate of 33% (Figure 1). Although this may be an overestimation, as not everyone was tested. All cases to date have been among passengers or crew on board the ship. Beyond the first 3 cases, 7 additional confirmed or probable cases were identified. A German national (case 4) died of a SARI on 2 May, and later testing in the Netherlands confirmed ANDV. Two crew members, the ship’s doctor (case 5) and an expedition guide (case 6), were medically evacuated to the Netherlands, tested positive for ANDV on 7 and 8 May respectively, and are in a stable condition.

On 22 April, case 7 disembarked on the island of St Helena mid-voyage, while still asymptomatic, before the hantavirus outbreak declaration, and returned to Switzerland. Following the ship operator’s notification, the patient presented to a Zurich hospital, was isolated and tested; the PCR test was positive for ANDV. Case 8, a passenger who disembarked in Tristan Da Cunha, developed symptoms on 28 April, is currently under medical care and considered a probable case due to sample and access to testing limitations. Two additional confirmed cases (France and Spain) were detected among individuals evacuated from the ship in Tenerife, Spain, who developed symptoms after disembarking on 10 and 12 May, respectively.

Early ANDV sequencing revealed high similarity to previously reported sequences from Argentina. An initial zoonotic introduction prior to the ship’s departure from Argentina is likely, as Case 1 developed symptoms on April 6 (departure date 1 April), and he had a 3-month travel history to regions with known ANDV enzoonotic circulation. No samples were available for this case. Sequencing of the subsequent cases showed a high level of genetic similarity, with a maximum of one detected SNP per individual^3^. Further epidemiological analysis is needed to decipher if this is a single or a limited number of zoonotic spillover events, or human-to-human transmission, as was seen in the 2018 outbreak, with similar clustering and lack of diversity^5^.

The situation is evolving, and WHO and ECDC assess the global risk as low. The primary source of the outbreak is under active investigation, with experts experienced with ANDV collaborating to focus on exposures to South American rodents prior to the cruise’s departure.

## Supplementary Material

Supplementary Appendix

## Figures and Tables

**Figure:**
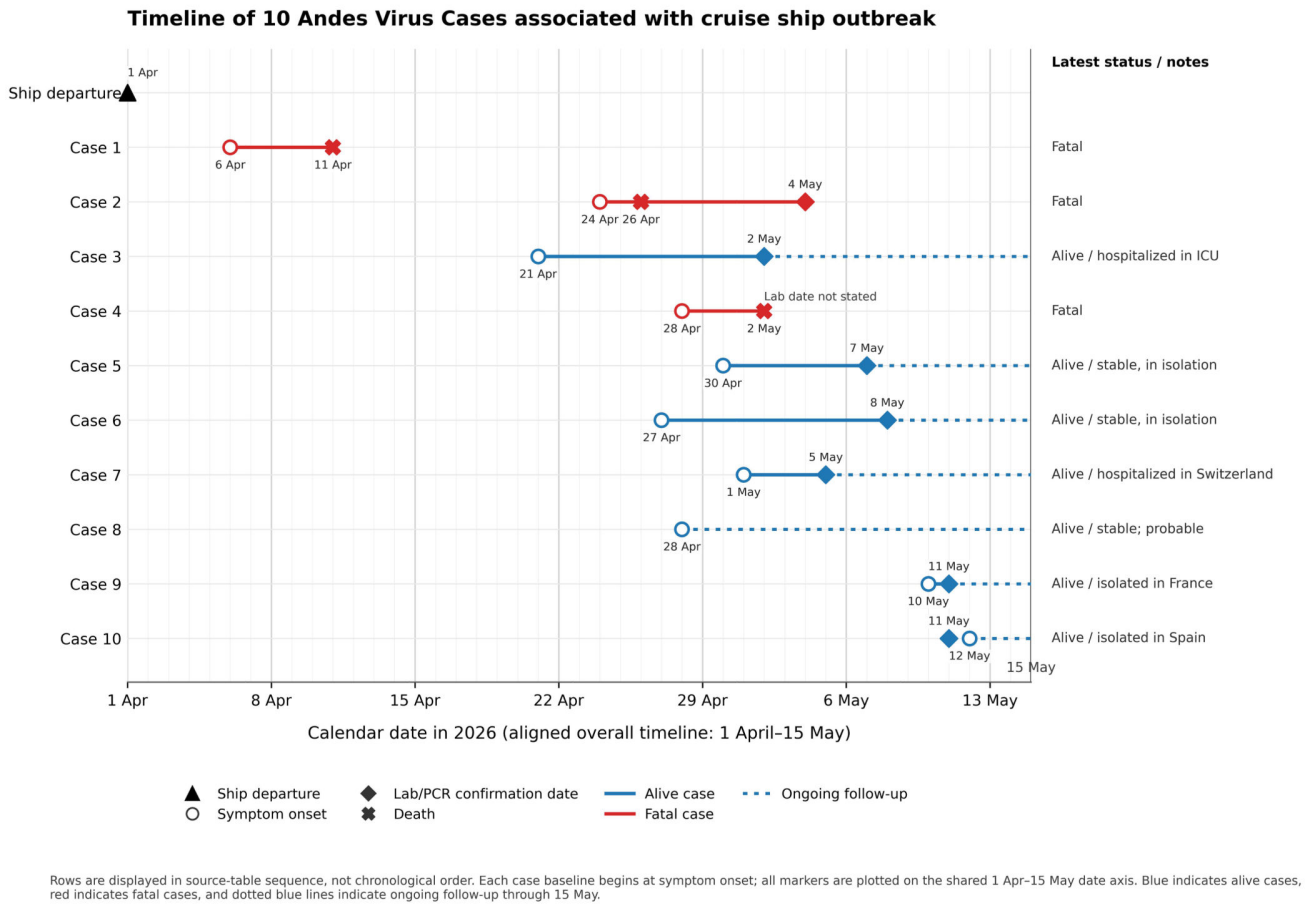
Timeline of the first 10 Andes virus cases associated with the MV *Hondius* cruise ship^2^
