# Supplementary material for: Andes Hantavirus Outbreak on a Cruise Ship, 2026
Source: N Engl J Med. Author manuscript; Available in PMC 2026 Jul 14. (PMC7619236; doi:10.1056/NEJMc2606496)
Supplement: Supplementary Appendix [file EMS216006-supplement-Supplementary_Appendix.pdf]

**Supplementary material**

# Andes Hantavirus Outbreak on a Cruise Ship, 2026

**Andes Virus Outbreak Working Group**

## Table of Contents

Author list in alphabetical order of surname: .......... 2
Brief background on Hantaviruses .......... 4
The differential diagnosis .......... 4
Initial microbiological investigations on case 3 .......... 4
Hantavirus testing and sequencing at NICD: .......... 6
Genomic sequences .......... 6
Passengers and crew on board the cruise ship .......... 6
Definition of high and low risk contacts[5] .......... 6
Outbreak management and coordination .......... 7
Acknowledgments: .......... 7
References .......... 8

## Author list in alphabetical order of surname:

| First name(s) | Last name | Affiliation | Qualification(s) |
|---|---|---|---|
| Leonidas | Alexakis | European Centre for Disease Prevention and Control | MD, MSc |
| Anna | Aryee | UK Health Security Agency | MBBS FRCPath |
| Agoritsa | Baka | European Centre for Disease Prevention and Control | MD, PhD |
| Tamas | Bakonyi | European Centre for Disease Prevention and Control | DVM,PhD, DSC |
| Freddy | Banza-Mutoka | WHO AFRO | MD, PhD |
| Marie Roseline | Belizare | World Health Organization | MD, PhD |
| Renu | Bindra | UK Health Security Agency | MBBS, FFPH |
| Lucille | Blumberg | National Institute for Communicable Disease, A Division of the National Health Laboratory Service | Mmed(Microbiology), DSc(Med) |
| Annelies | Brilman | National Institute for Public Health and the Environment | MD PhD |
| Colin | Brown | UK Health Security Agency | FRCPath, FFPH |
| Orlando | Cenciarelli | European Centre for Disease Prevention and Control | PhD, MSc |
| Meera | Chand | UK Health Security Agency | MBBS, FRCPath |
| Menno | De Jong | National Institute for Public Health and the Environment | MD PhD |
| Sacha | de Stoppelaar | National Institute for Public Health and the Environment | MD PhD |
| Mathew | Dryden | UK Health Security Agency | MD FRCPS |
| Shenaaz | El-Halabi | World Health Organization | MPH |
| Nevashan | Govender | National Institute for Communicable Disease, A Division of the National Health Laboratory Service | MSc, MPH |
| Esther | Hamblion | World Health Organization | PhD, MSc |
| William | Hardy | Ascension Island Government | MD |
| Andreas | Hoefer | European Centre for Disease Prevention and Control | PhD, MSc |
| Thomas | Hofmann | European Centre for Disease Prevention and Control | MHE, MPH |
| Ana | Hoxha | World Health Organization | PharmD MSc |
| Chikwe | Ihekweazu | World Health Organization | FFPH, MPH |
| Thomas | Inns | UK Health Security Agency | PhD, FFPH |
| Nazir Ahmed | Ismail | National Institute for Communicable Disease, A Division of the National Health Laboratory Service | MBChB, FCPath(SA) |
| Kuban | Iyer | Mediclinic Sandton | MBBCh, FCP(SA) |
| Hilary | Kirkbride | UK Health Security Agency | MBChB, FFPH |

| | | | |
|---|---|---|---|
| Vuyiswa | Kumalo | National Institute for Communicable Disease, A Division of the National Health Laboratory Service | MBChB, DipPEC(SA) |
| Favelle | Lamb | European Centre for Disease Prevention and Control | Lic, MSc |
| Olivier | le Polain de Waroux | World Health Organization | MD PhD |
| Tjalling | Leenstra | National Institute for Public Health and the Environment | MD PhD |
| Anais | Legand | World Health Organization | MPH |
| Abdi Rahman | Mahamud | World Health Organization | MD |
| Terry | Marshall | Ampath Laboratories | MB.BCh, FCPath(SA) |
| Grazina | Mirinaviciute | European Centre for Disease Prevention and Control | PhD, MPH |
| Chanelle | Moore | Ampath Laboratories | MB.BCh, FCPath(SA) |
| Jeremy | Nel | University of the Witwatersrand | MBChB, MSc (Epi) |
| John | Otshudiema | World Health Organization | MD, MPH |
| Boris I | Pavlin | World Health Organization | MD MPH |
| Richard | Pebody | UK Health Security Agency | MRCP, FFPH |
| Ihor | Perehinets | World Health Organization | MD |
| David | Peres | European Centre for Disease Prevention and Control | MD, MPH |
| Richard | Puleston | UK Health Security Agency | MBChB, FFPH |
| Otim Patrick | Ramadan | World Health Organization | MBChB, MPH |
| Chantal | Reusken | National Institute for Public Health and the Environment | PhD |
| Adriana | Romani Vidal | European Centre for Disease Prevention and Control | MD, MPH |
| Wilhelmina L. M. | Ruijs | National Institute for Public Health and the Environment | MD PhD |
| Esther | Schadd | Utrecht Military Hospital | MD |
| Dubravka | Selenic Minet | World Health Organization | DVM, MSc |
| Ettore | Severi | European Centre for Disease Prevention and Control | PhD, MSc |
| Evan | Shoul | University of Witwatersrand | MBChB; FCP(SA) |
| Lerato | Sikhosana | National Institute for Communicable Disease, A Division of the National Health Laboratory Service | MBChB, FCPath(SA) |
| Fernando | Simón | Ministerio de Sanidad | MD |
| Gianfranco | Spiteri | European Centre for Disease Prevention and Control | MD, MSc(Public Health) |
| Lorenzo | Subissi | World Health Organization | MSc, PhD |
| Margreet | te Wierik | National Institute for Public Health and the Environment | MD, PhD |

| | | | |
|---|---|---|---|
| Sherine | Thomas | UK Health Security Agency | MBChB, MRCP(ID) |
| Annemiek A. | Van der Eijk | University Medical Center Rotterdam | MD PhD |
| Maria | Van Kerkhove | World Health Organization | MS, PhD |
| Michele | van Vugt | Amsterdam UMC | MD, PhD |
| Thijs | Veenstra | National Institute for Public Health and the Environment | MSc |
| Veronique | Verhoeven | University of Antwerp | MD, PhD |
| Albert | Vollaard | National Institute for Public Health and the Environment | MD PHD |
| Joseph Francis | Wamala | World Health Organization | MD, PhD |
| William | Welfare | UK Health Security Agency | MBChB, FFPH |
| Jacqueline | Weyer | National Institute for Communicable Disease, A Division of the National Health Laboratory Service | PhD, MPH |

## Brief background on Hantaviruses

Most hantaviruses are not transmitted from human to human (H-H). In general, they are acquired through contact with the excreta of infected rodents. Orthohantavirus andense (ANDV) has an enzootic circulation in certain rodent populations in Argentina, Chile, Brazil, Uruguay, Paraguay and Bolivia[1,2]. It is the only hantavirus with H-H transmission among close contacts. HCPS clinical features begin with non-specific symptoms, including fever and gastrointestinal disturbances, followed 3-5 days later by a cardio-pulmonary phase that can develop rapidly over 1-2 days, with a case fatality rate of 30-50%[3].

## The differential diagnosis

The differential diagnosis in this situation is very broad and includes atypical pneumonias such as Covid-19, influenza, particularly avian influenza, which had been circulating in South America, psittacosis (given a history of birdwatching), and legionellosis. Additional possibilities included bacterial or fungal sepsis with ARDS, malaria, tick-bite fever and dengue, which is also endemic in South America.

## Initial microbiological investigations on case 3

- Nasopharyngeal Sample and Tracheal Aspirate
    - Seegene:
        - Allplex Respiratory panel 1A (reports Influenza A and types to H1, H1pdm09, or H3. RSV A and RSV B. There is an exogenous internal control)

        - Allplex respiratory panel 2 (reports enterovirus, Adenovirus, hMetapneumovirus, human Parainfluenza viruses 1, 2, 3, 4. There is an exogenous internal control).
        - Allplex respiratory panel 3 (reports bocavirus, coronavirus 229E, coronavirus NL63, coronavirus OC43, rhinovirus. There is an exogenous intrernal control)
        - Allplex Pneumobacter panel (reports Bordetella parapertussis, Bordetella pertussis, M.pneumoniae, C.pneumoniae, H.influenzae, S.pneumoniae, Legionella pneumophila. There is an exogenous internal control)
        - Allplex SARS-CoV-2 Assay
    - All results negative
- Tracheal aspirate only
    - BioGX on the BD Max platform, which tests for M.pneumoniae, C.pneumoniae, Legionella spp, and C. psittaci.
    - BioFire FILMARRAY pneumonia panel that tests semiquantitative results for multiple bacterial causes of pneumonia, viruses, and, of importance in this patient, Legionella pneumophila again, and some resistance genes.
    - All of the above results were negative, with the exception of a S.pneumoniae $10^4$, which most likely represented colonization rather than the cause of the diffuse pulmonary infiltrates seen on chest imaging of this patient.
- Urine
    - A urine Legionella antigen test was also performed and was negative.
- Blood cultures
    - Blood cultures sets from 30 April and 1 May were negative
- Sputum
    - Microscopy, culture and sensitivity: No pathogens identified from the 30 April
- Nostril, groin, armpit swabs
    - Negative for S.aureus.
- Serology for Dengue
    - IgG positive, IgM negative - likely a previous infection
- Cerebrospinal fluid
    - No growth, no bacterial or fungal cells seen,
    - Qiastat ME panel all negative and
    - No cells.
    - Hantavirus PCR negative.
- Fungitel: negative

- Malaria smears and antigen
    - Negative

## Hantavirus testing and sequencing at NICD:

Reverse transcriptase PCR: Nucleic acid was extracted from whole blood and serum specimens using the QiaQube Viral RNA minikit (Qiagen, Germany) according to the manufacturer's instructions. A nested RT-PCR assay adapted from Klempa et al was used[4]. The assay targets the highly conserved L-segment of hantaviruses.

## Genomic sequences

Details with analysis: https://virological.org/t/preliminary-analysis-of-orthohantavirus-andesense-virus-sequences-from-a-cruise-ship-related-cluster-may-2026/1029

Sequences: pathoplexus (https://pathoplexus.org/) with accession numbers: PP_006WDJK.1 (12 May 2026) and PP_006WDKH.1 (12 May 2026)

## Passengers and crew on board the cruise ship

The passenger manifest from the cruise ship company listed 149 individuals: 88 passengers and 61 crew, a slight variation from the initial report. 2 crew members and 1 passenger were medically evacuated on 6 May.

## Definition of high and low risk contacts[5]

High-risk contacts:

Individuals with one or more of the following exposures with a probable or confirmed ANDV case: • Persons sharing the same cabin. • Intimate partners or individuals with direct physical contact. • Persons sharing a bathroom or sleeping space. • Persons within approximately 2 meters for prolonged periods (>15 minutes cumulative) indoor. • Persons participating in shared meals, prolonged social interactions, or caregiving activities. • Healthcare workers with unprotected exposure. • Healthcare workers exposed without appropriate PPE during aerosol-generating medical procedures. • Aircraft passengers seated in the same row, and within two rows in all directions from the case. • Cabin crew or transport staff with interaction with the case. • Persons handling linens, clothing, other personal items of the case, medical waste, or body fluids without appropriate PPE

Low-risk contacts:

Individuals who have attended an event, been in a conveyance with a probable or confirmed ANDV case but have no known direct or prolonged close interaction with the case, including: • Other passengers or crew without cabin sharing or prolonged close interaction on a ship. • Aircraft passengers outside the defined seating proximity zone. •

Brief transit or port contacts not meeting the high-risk contact definition. • Individuals sharing large open-air spaces without prolonged interaction. • Healthcare providers using appropriate PPE throughout exposure.

## Outbreak management and coordination

The outbreak is being managed through an international response coordinated by WHO under the IHR in collaboration with ECDC and Dutch authorities and includes in-depth epidemiological investigations, case isolation and care, medical evacuation, laboratory investigations and an operation to repatriate passengers and crew to their home countries, where they will be quarantined and monitored. Those unable to return to their home countries are in quarantine facilities in the Netherlands under active monitoring. Currently, management of this public health event has been initiated in Cabo Verde, France, the Netherlands, Spain, SA and the UK, with support from the WHO and the ECDC. Contact tracing has been initiated for all potentially exposed individuals, including those on the cruise ship, undertakers, healthcare workers on the air ambulance, healthcare facilities and contacts of cases who were on commercial aircraft from St Helena to Johannesburg and from Johannesburg to Amsterdam.

Experts from the WHO and the European Center for Disease Prevention and Control (ECDC), along with two infectious disease physicians from the Netherlands, boarded the ship in Cabo Verde on 6 May to conduct medical examinations and collect detailed exposure histories of passengers and crew.

The response has been swift across countries, with information shared through IHR, EU early warning systems and convened expert networks for clinical management, infection prevention and control and laboratory and technical guidance from WHO and ECDC. To limit further spread and due to uncertainties, WHO and ECDC consider all passengers and crew on board to be high-risk contacts. All others are risk-stratified as high- or low-risk, and definitions are provided below. A detailed analysis of exposure levels and infection risk among people on board, as well as among passengers who disembarked in St Helena, has been undertaken. Uncertainty about person-to-person transmission during the prodrome and reports of “superspreading” within crowded environments in a previous ANDV outbreak[6,7] have led to the application of quarantine to high-risk contacts, as this strategy has been shown to reduce the reproductive number (Rn). Active follow-up to monitor symptoms is required with immediate self-isolation or isolation in a medical facility if symptoms develop.

## Acknowledgments:

We thank all the staff involved in the multi-country and multi-institutional efforts.

The first draft of Figure 1 was generated using ChatGPT based on a prompt referencing data on cases from: https://www.who.int/emergencies/disease-outbreak-news/item/2026-DON600 and further refined based on additional information sourced for this paper.

## References

1. Martinez VP, Bellomo C, San Juan J, et al. Person-to-person transmission of Andes virus. *Emerg Infect Dis* 2005; **11**(12): 1848-53.
2. UKHSA. Andes hantavirus: epidemiology, outbreaks and guidance. 2021. https://www.gov.uk/guidance/andes-hantavirus-epidemiology-outbreaks-and-guidance (accessed 10 May 2026.
3. Vial PA, Ferrés M, Vial C, et al. Hantavirus in humans: a review of clinical aspects and management. *The Lancet Infectious Diseases* 2023; **23**(9): e371-e82.
4. Klempa B, Fichet-Calvet E, Lecompte E, et al. Hantavirus in African wood mouse, Guinea. *Emerg Infect Dis* 2006; **12**(5): 838-40.
5. WHO. Management of contacts of Andes virus (ANDV) cases from the MV Hondius cruise ship. 2026. https://www.who.int/publications/m/item/management-of-contacts-of-andes-virus-(andv)-cases-fromthe-mv-hondius-cruise-ship (accessed 15 May 2026.
6. Martínez VP, Di Paola N, Alonso DO, et al. "Super-Spreaders" and Person-to-Person Transmission of Andes Virus in Argentina. *N Engl J Med* 2020; **383**(23): 2230-41.
7. Alonso DO, Pérez-Sautu U, Bellomo CM, et al. Person-to-Person Transmission of Andes Virus in Hantavirus Pulmonary Syndrome, Argentina, 2014. *Emerg Infect Dis* 2020; **26**(4): 756-9.
